# Prognostic correlations with the microbiome of breast cancer subtypes

**DOI:** 10.1038/s41419-021-04092-x

**Published:** 2021-09-04

**Authors:** Sagarika Banerjee, Zhi Wei, Tian Tian, Dipayan Bose, Natalie N. C. Shih, Michael D. Feldman, Thaer Khoury, Angela De Michele, Erle S. Robertson

**Affiliations:** 1 Department of Otorhinolaryngology-Head and Neck Surgery, Perelman School of Medicine, University of Pennsylvania, Philadelphia, PA USA; 2 Department of Computer Science, New Jersey Institute of Technology, Newark, NJ USA; 3 Department of Pathology and Laboratory Medicine, Perelman School of Medicine, University of Pennsylvania, Philadelphia, PA USA; 4 Department of Pathology, Roswell Park Cancer Institute, Buffalo, NY USA; 5 Division of Hematology Oncology, Department of Medicine, Perelman School of Medicine, University of Pennsylvania, Philadelphia, PA USA

**Keywords:** Cancer, Prognostic markers

## Abstract

Alterations to the natural microbiome are linked to different diseases, and the presence or absence of specific microbes is directly related to disease outcomes. We performed a comprehensive analysis with unique cohorts of the four subtypes of breast cancer (BC) characterized by their microbial signatures, using a pan-pathogen microarray strategy. The signature (includes viruses, bacteria, fungi, and parasites) of each tumor subtype was correlated with clinical data to identify microbes with prognostic potential. The subtypes of BC had specific viromes and microbiomes, with ER+ and TN tumors showing the most and least diverse microbiome, respectively. The specific microbial signatures allowed discrimination between different BC subtypes. Furthermore, we demonstrated correlations between the presence and absence of specific microbes in BC subtypes with the clinical outcomes. This study provides a comprehensive map of the oncobiome of BC subtypes, with insights into disease prognosis that can be critical for precision therapeutic intervention strategies.

## Introduction

The microbiome can influence many aspects of normal healthy life and specific changes may have clinical implications for several diseases [1–5]. Furthermore, specific microbial signatures are associated with different types of cancers [2, 3, 6–10]. Therefore, we posit that pathological states, like oncogenesis, create microenvironments amenable to the persistence of a disease-specific microbiome. Alternatively, a pre-existing microbiome in a microenvironment may contribute to the development of the disease. Therefore, disease-specific microbiome may have prognostic and diagnostic value. In addition, the cancer microbiome (oncobiome, inclusive of viruses, bacteria, fungi, and parasites) and its metabolites have a major impact on the local and distant immune system, which can influence clinical outcomes in cancer patients [11, 12].

There are four subtypes of breast cancer (BC) that are based on the status of the estrogen receptor, progesterone receptor, and human epidermal growth (Her2) expression in cancerous breast cells [3]. The endocrine hormone receptor positive cancers include (1) estrogen receptor positive and/or progesterone receptor positive, and Her2 negative (designated herein as ER), and (2) triple positive (TP) cancers that are ER positive, PR positive, and Her2 positive [13]. These cancers are generally responsive to treatment with hormone receptor blockers [13]. They are less aggressive with better prognosis compared to hormone receptor negative BCs, which include the Her2 BC (Her2+, ER–, and PR–, designated herein as HR), and triple-negative (TN) cancer, which are ER, PR, and Her2 negative [9, 14]. TN BC (15–20% of BC patients) is the most aggressive of all the BCs, is non-responsive to treatment, is highly angiogenic, highly proliferative, and has the lowest survival rate [15].

Identifying the oncobiome of the four BC subtypes may identify a connection between the microbiome and therapeutic response to treatment [12, 16]. Recent studies have shown that the status of the microbiome may improve response to cancer therapies [11, 12]. In the present study, we used the pan-pathogen microarray (PathoChip [17]) to screen a larger cohort of BC and control patient samples, to validate our previous small-scale study [3], and showed a trend or correlation between unique microbial signature patterns in different BC types with clinical intervention or outcomes. This could provide both prognostic and diagnostic values for BC subtypes. Our findings demonstrated that the oncobiome of each BC cancer subtype is diverse and contains a variety of microbial signatures. ER showed the most diverse oncobiome, while TN was the least diverse. Further, each BC subtype can be distinguished by the presence or absence of specific viruses and other microbes, and thus the level of detection of these microbes was predictive of patient outcomes. Our data suggest that a thorough knowledge of the status of the tumor oncobiome is important and provides prognostic and diagnostic information toward precision patient care.

## Materials and methods

All experiments were performed according to relevant guidelines and regulations, and according to all the licensing and approvals by institutional committees at Perelman School of Medicine, University of Pennsylvania and the University of Buffalo School of Medicine, Roswell Park Cancer Institute IRB# BDR084317 who provided an independent cohort of patients and clinical data to support our findings.

### PathoChip design

The details of the PathoChip array have been previously described in detail [18]. The PathoChip contains 60,000 probes for parallel DNA and RNA detection of viruses (>4200), and known pathogenic bacteria (>320), fungi (>360), helminths (>250), and protozoa (>130) [17]. The array contains two types of probes: unique probes for each virus and other microorganism, and conserved probes that target genomic regions conserved between members of a family of viruses. The conserved probes allow detection of detect previously uncharacterized members of the family. These bespoke arrays are SurePrint glass slide microarrays (Agilent Technologies Inc.), containing eight replicate arrays per slide. Each probe is a 60 nucleotide (nt) DNA oligomer that targets genomic regions of viruses, prokaryotic, and eukaryotic microorganisms [3, 7, 18, 19].

### Sample preparation and microarray processing

Cohort of 95–105 formalin-fixed paraffin-embedded (FFPE) samples for each BC subtypes, 20 matched control samples, and 68 non-matched control samples from breast reduction surgeries were received as 10 µm sections. The de-identified samples we obtained as an independent cohort for each BC subtype to validate and provide clinical data of prognostic value. IRB approval was obtained from the Roswell Park Cancer Institute Internal Review Board. The Biomedical Data Science office delivers HIPAA compliant de-identified clinical data that ensures IRB compliance as Institute Honest Brokers. Patient identifiers were stripped from all data files and replaced with a de-identified ID. The Biomedical Data Science Office staff is the holder of the identified information. Researchers are unable to match patient samples and clinical data back to the identified patient information. HIPAA compliant de-identified patient samples and clinical data were delivered to University of Pennsylvania. Consequently, we obtained clinical information for these samples, including age of the patients, grade, stage of the tumor, primary site of the tumor, age at diagnosis, recurrence type, response to treatment, survival and disease-free time post treatment. The tumor and control tissues were prepared, examined, and verified, by the breast pathologists at the Department of Pathology, RPCI, Buffalo, New York. The samples were prepared and cut in a sterile environment and the microtome sterilized between samples, to prevent contamination between sample. Utmost care was taken during the procurement and handling of the samples, and during PathoChip screening to minimize contamination.

The PathoChip screen workflow was described previously [3, 6–8]. Briefly, DNA and RNA were extracted from FFPE samples; 50 ng each of DNA and 50 ng of RNA were used for whole transcriptome amplification using the TransPlex Whole Transcriptome Amplification Kit (Sigma-Aldrich, St. Louis, MO). Human reference RNA and DNA were extracted from the human B cell line, BJAB (obtained from ATCC, and cultured in the lab for less than 6 months) and 15 ng of each were used for WTA. The cellular DNA/RNA provided a reference to compensate for dye bias. One microgram of amplified products from the cancer and control tissues was labeled with Cy3, and the human reference was labeled with Cy5 (SureTag labeling kit, Agilent Technologies, Santa Clara, CA). The labeled samples (Cy3 plus Cy5) were hybridized to the PathoChip for 40 h at 65 °C with rotation. The slides were then washed and scanned for visualization using an Agilent SureScan G4900DA array scanner.

### Microarray data extraction and statistical analysis

The microarray data extraction and analyses have been described previously [3]. Raw data from the images were extracted with Agilent Feature Extraction software. We used the R-program for normalization and data analyses [20, 21]. The microarray data are available in Gene Expression Omnibus. We calculated scale factors using signals of green and red channels for human probes. Scale factors are the sum of green and sum of red signal ratios [∑(g)/∑(r)] of human probes. Then we used scale factors to obtain normalized signals for all other probes. For all probes except human probes, normalized signal is log2 transformed of green signals/scale factors modified red signals (log2g-scale factor*log2r). On the normalized signals, *t*-test was applied to select probes present in cancer samples by comparing cancer samples versus controls and to select probes present in the BC samples versus the non-matched controls. The cut-off for significant detections in cancers versus the controls was log2 fold change >1 and adjusted *p* value (with multiple testing corrections) <0.05. Prevalence was calculated by counting the number of cancer cases with hybridization signals greater than the average signal or negative control probes and represented as a percentage.

Analyses at the individual probe level (both for specific and conserved probes), and at the family (for viruses) or genera (for bacteria, fungi, and parasite) level, taking into account all the probes per family or genera, were performed. Microbial detections were represented based on their average hybridization signal (average of the hybridization signals of detected probes per family or genera) and prevalence.

The cancer samples were also subjected to unsupervised hierarchical clustering, based on the detection of microbial signatures in the samples (average hybridization signal per viral family or microbial genus), using the R-program (Euclidean distance, complete linkage, non-adjusted values) [21, 22].

**Fig. 1:**
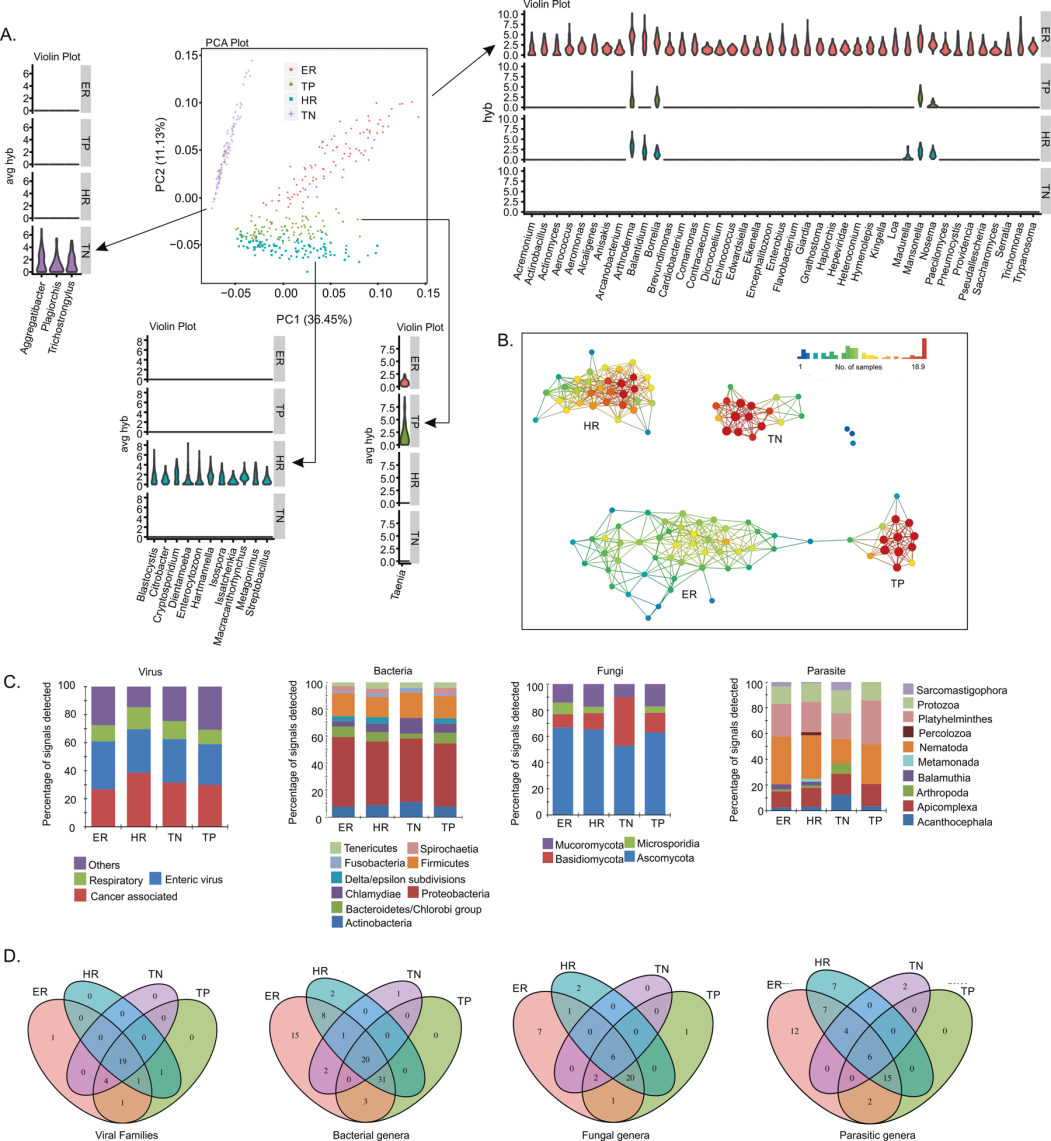
Oncobiome diversity in different breast cancer subtypes. **A** The four types of breast cancers have distinct oncobiome compositions. PCA plot using NBClust shows that TN breast cancer oncobiome is strikingly different from the other three breast cancer subtypes studied. The unique aspects of the oncobiomes of each breast cancer subtype are represented as violin plots showing the full distribution of the data. ER+ BC shows the most diversity in oncobiome. **B** Using topological data analysis, we further show the similarity in the oncobiomes of triple positive and ER positive BCs, while both Her2+ and triple-negative breast cancer have oncobiome characteristics very different from other BCs. **C** Bar graphs showing different types and phyla of oncobiome in the four breast cancer subtypes. **D** Venn diagrams show the viral and microbial signatures that are shared and unique to the four breast cancer subtypes.

After obtaining the aggregated hybridization signals (average of the hybridization signals of probes from same family or genus; for virus, we aggregated per family; for bacteria, fungi, and parasite, we aggregated per genus) of the oncobiome of each BC subtype, we used principal component analysis (PCA) plot to display the four BC subtypes of BCs (Fig. 1A). Violin plots were used to display the distinct microbial signatures, i.e., the organisms that were detected in one BC subtype only; or the organisms that had significantly higher aggregated hybridization signals in one subtype compared to the other three (one-sided Wilcoxon test *p* value < 0.05 and logFC > 1) (Fig. 1A).

Additional topological-based data analyses were conducted with Ayasdi software (Ayasdi, Inc.) using correlation metric, and metric PCA co-ordinates lenses (Fig. 1B). The differences in microbial detections between different types of BCs were determined using the two-sided *t*-test (Supplementary Table S2).

### Clinical analysis in each cancer subtypes

We first choose organisms that had correlation with disease outcomes. Based on the median of hybridization signals of each organism, we divided patients into high and low groups. We applied Kaplan–Meier survival analysis [23] to test if the survival rates or disease-free rates were significantly different in the high and low groups. We applied the Benjamini–Hochberg procedure [24] for multiple testing correction. No organisms had adjusted *p* values < 0.05 (Supplementary Material S8), and we reported the top ones with a nominal *p* value < 0.05 to highlight the trend. We also ran Cox regression [25] with prognostic factors included for testing disease outcome association. See Supplementary Table S8 for the detailed Cox regression results. Then patients were subjected to clustering based on the disease outcomes correlated organisms. Since the prevailing zero measurements, we applied a robust multi-kernel clustering method—SIMLR [21] to group patients into two clusters (the number of clusters was determined by the “SIMLR_Estimate_Number_of_Clusters” function). Barplot and heatmap were made to display the proportions of clinical features and hybridization signals of organisms, respectively. To gain more statistical power, we aggregated numerical clinical features into levels. The tumor sizes were aggregated into three levels: T1 (<20 mm), T2 (20–50 mm), and T3 (>50 mm). The diagnosis ages were aggregated into two levels: ≤40, and >40, since age 40 is a critical age as women over age 40 have increased rates of BC [26]. *χ*^2^ test was conducted to compare if there were significant differences in proportions of clinical features between the two clusters. To compare the proportions of some interesting clinical features (e.g., stage 3–4, distant metastasis, etc.), we used one-sided Fisher’s exact test. We also applied cox regression to see if clinical factors (such as tumor size, and grade) were correlated with survival and disease-free rates (Supplementary Table S9).

## Results

### Microbiome characteristics in different subtypes of breast cancers

**Fig. 2:**
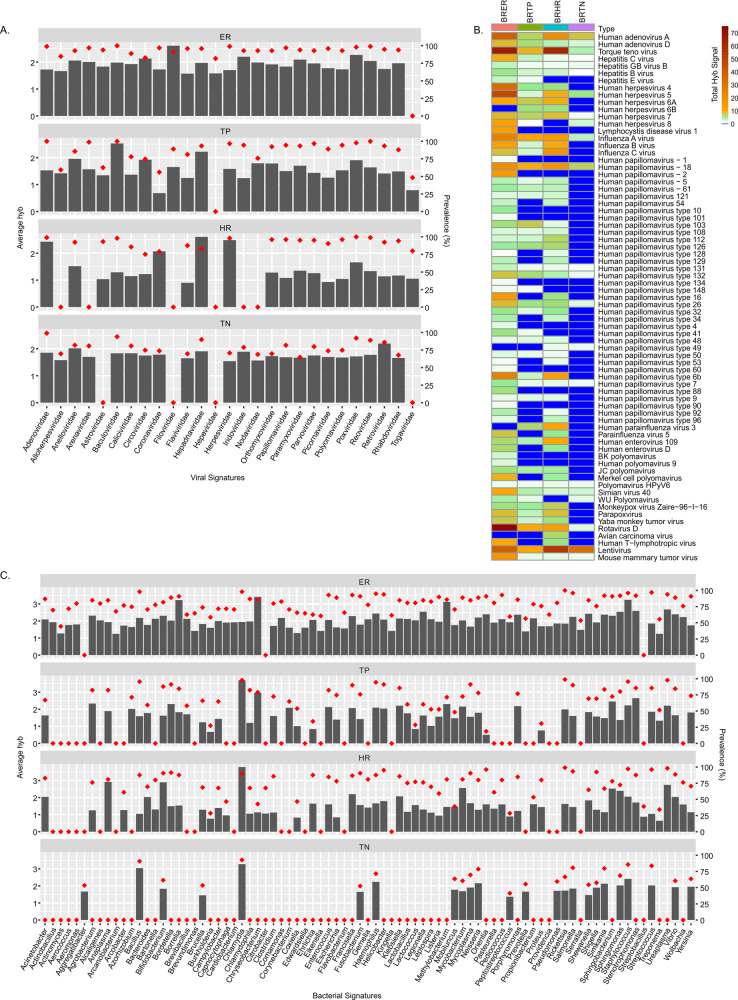
Viral and bacterial microbial signatures detected in the four types of breast cancers. **A**, **C** The bars represent the average hybridization signal for each virus and bacterial signatures respectively, while the percent prevalence of those virus and bacterial signatures in the sample set is indicated by the red dots. **B** The average hybridization signals for specific viruses were summed and represented as heatmap to show low to high detections of specific viral signatures in the four types of breast cancers.

**Fig. 3:**
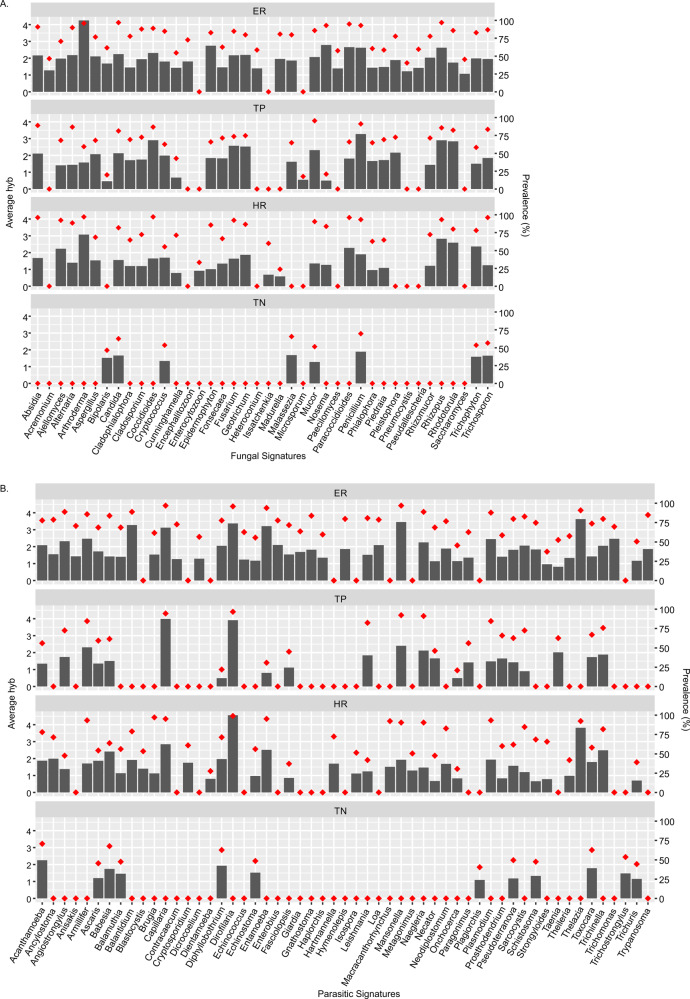
Fungal and parasitic signatures detected in the breast cancers. **A**, **B** The bars represent the average hybridization signal for each fungal or parasitic signatures, while the percent prevalence of those fungal or parasitic signatures in the sample set is indicated by the orange dots.

Microarray analysis was performed to identify the oncobiome of four different BC subtypes (shown in Figs. 1–3). The PCA of on the oncobiomes of the four BC subtypes (Fig. 1) validated our previous study [3]. The TN BC oncobiome was strikingly different from the others. This was primarily due to (1) the detection of fewer microbial agents in the TN samples (the least diverse); (2) a significantly higher detection of *Aggregatibacter* (Fig. 1A, violin plots); and (3) the detection of *Plagiorchis* and *Trichostrongylus* (Fig. 1A, violin plots). These factors made a distinct cluster for the TN BC samples in the PCA plot. Conversely, ER+ BC samples showed the most robust oncobiome, with a greater number of bacterial (mostly Proteobacteria), fungal, viral, and parasitic signatures with higher unique signals (Fig. 1A, violin plot). HR and TP BC subtypes showed intermediate oncobiome densities with fewer bacterial (*Citrobacter*, *Streptobacillus*), fungal (*Enterocytozoon*, *Issatchenkia*), and parasitic (*Blastocystis*, *Cryptosporidium*, *Dientamoeba*, *Hartmannella*, *Isospora*, *Macracanthorhynchus*, *Metagonimus*) signatures uniquely detected, or had significantly higher signals in the HR samples. The parasitic signature of *Taenia* was prominently detected in TP samples.

Figure 1B used topological data analysis that suggested greater similarity between the oncobiomes of TP and ER subtypes, while HR and TN cancers had oncobiome characteristics very different from the other BCs. Among the receptor negative BCs, HR differed from TN by having greater signals for signatures of *Togaviridae* and *Astroviridae*, and signatures of *Ehrlichia*, *Wolbachia*, *Bartonella*, *Legionella*, and *Campylobacter*, whereas the TNs had higher signals for signatures of *Alloherpesviridae*, *Arenaviridae*, and *Nodaviridae* compared to the HRs (Supplementary Table S2). Among the ERs had higher signals for signatures of *Hepeviridae*, *Aeromonas*, *Alcaligenes*, *Propionibacterium*, *Capnocytophaga*, *Pediococcus*, *Bartonella*, *Pasteurella*, *Madurella*, and *Ancylostoma* compared to the TPs (Supplementary Table S2). The receptor positives (ER and TP) as a whole had higher signals for signatures of *Filoviridae*, *Pleistophora*, *Azorhizobium*, *Paragonimus*, *Taenia*, *Corynebacterium*, *Brevibacillus*, *Chryseobacterium*, *Angiostrongylus*, and *Leishmania* (Supplementary Table S2).

Figure 1C shows the overall percentage of signatures (viral, bacterial, fungal, and parasite) in each BC subtype separated into different types and phyla. In the oncobiomes of the four BC subtypes, we found that cancer-associated viral signatures made up 26 and 38% of the total viral signatures, with the highest percentage in HR (Fig. 1C, virus). Enteric viruses represented an equal proportion in each BC subtype, while respiratory viruses made up 10–15%, and a variety of other viruses made up the remainder of each virome (Fig. 1C and Supplementary Table S1, virus).

The bacterial signatures of all the BC subtypes screened were predominated by Proteobacteria (40–50%), followed by a lower percentage of Firmicutes and small amounts of a variety of other bacterial types (Fig. 1C, bacteria).

The fungal signatures in the oncobiome of the four BC subtypes were predominated by the phylum Ascomycota (50–60%, Fig. 1C, fungi); however, the TN oncobiome included a higher percentage of Basidiomycota compared to the other BC subtypes.

The parasitic signatures in the oncobiome of the four BC subtypes generally had a higher percentage of Nematoda, followed by Platyhelminthes, Apicomplexa, and Protozoa (Fig. 1C, parasites). HR samples had the most diverse parasitic presence followed by ER positive with TN having the least diversity.

Figure 1D shows Venn diagrams displaying the viral and other microbial signatures that are shared and unique to the four BC subtypes. Signatures of 19 viral families, 20 bacterial genera, 6 fungal genera, and 6 parasitic genera were detected in all the four BC subtypes (Fig. 1D and Supplementary Table S10). ER and TP cancers shared 1 viral family signature, 3 bacterial genera, 1 fungal genus, and 2 parasitic genera. HR and TN cancers shared no viral, bacterial, fungal, or parasite signatures. A few viruses and other microbes were found to be unique to each of the four BC subtypes and they are better highlighted in Fig. 2 and Supplementary Table S10.

Together the data in Fig. 1 showed the broad diversity of viruses and other microbes that make up the BC oncobiome. Yet within this diversity, the oncobiome of each BC subtype had unique characteristics that make them distinguishable.

### Viral and other microbial signatures, and their prevalence in the four breast cancer subtypes

In Figs. 2A, C and 3A, B, the bar graphs indicate the average hybridization signal for different families of viral and other microorganisms detected in the four BC subtypes. The red diamonds indicate the percent prevalence of the viruses and microorganisms in each BC subtype.

Figure 2A shows that a variety of viral families are detected at varying hybridization intensities in each BC subtype. ER and HR tumors had the most and the least diverse virome. The other BC subtypes lacked specific viral families, suggesting that the different BC subtype can be distinguished by the presence or absence of signatures for specific viral families.

Figure 2B shows a heatmap of the total hybridization signals for viruses represented in the four different BC subtypes. Interestingly, TN cancer showed little to no papilloma signal except for a very low detection of HPV 18 and even lower signals for HPV 7, 26, 49, 131, and 132. The ER subtype showed low to moderate levels of papillomaviruses except for HPV49. The oncogenic HPV16 was detected only in ER and HR subtypes, while oncogenic HPV 18 was detected at low to moderate levels in all BC subtypes. Other oncogenic viruses specifically the adeno and hepatitis viruses were seen in all BC subtypes, HHV8 primarily in ER and TN, Merkel Cell Polyoma Virus and human T-lymphotropic virus (HTLV) in both ER and HR. Interestingly, signatures related to oncogenic viruses that are better characterized in non-human hosts were also detected. For example, Yaba Monkey Tumor Virus and Monkeypox Zaire were detected in three BC subtypes, Avian Carcinoma Virus signature in HR, Mouse Mammary Tumor Virus detected in all, Torque Teno Virus detected in all, and Parapoxvirus detected in three subtypes except TN. These signatures may indicate human variants of these viruses. In this regard, a signature related to HIV-1 sequences was also detected with high average hybridization signal in all the BC subtypes. Since these patients were HIV negative, this result suggests the probable presence of an uncharacterized human lentivirus.

Figure 2C shows the representation and prevalence of bacterial genera in the BC subtypes. ER subtype had the most diverse bacterial microbiome, whereas TN subtype had a modest bacterial microbiome. Some of the bacterial signatures were detected with high average hybridization signal intensity, suggesting higher levels of nucleic acids representing these bacteria (Fig. 2C). For example, we noted higher detection of *Bacillus* and *Chlamydia* in 90% of TN samples; *Chlamydia*, *Anaplasma*, and *Bifidobacterium* in 80–90% of HR samples; *Chlamydia* and *Chrysobacterium* in 82–98% of TP samples; and *Borrelia, Chrysobacterium, Methylobacterium*, and *Staphylococcus* in 85–95% of ER samples (Fig. 2C and Supplementary Table S1). Among the genera in the BC subtypes those in the phylum proteobacteria dominated (approx. 55%) followed by Firmicutes and Actinobacteria (Fig. 1C and Supplementary Table S1). These include *Brucella*, *Haemophilus*, *Neisseria*, *Rickettsia*, *Salmonella*, *Shewanella*, *Shigella*, *Sphingomonas*, *Vibrio*, and *Yersinia* from the proteobacteria (Fig. 2C and Supplementary Table S1). The Bacteroidetes phyla were next predominant. However, we detected more Chlamydiae followed by the Bacteroidetes in the TNs (Fig. 1C and Supplementary Table S1). Tenericutes and Fusobacteria were also detected in all BC subtypes (Fig. 1C and Supplementary Table S1).

### Specific fungal and parasite signatures and their prevalence in the four breast cancer types

Each BC subtypes had unique fungal signatures (Fig. 3A). The most diverse fungal biome (mycobiome) was detected in ER and the least complex in TN. Most of the fungal signatures detected in the TN samples were yeast or skin fungi and were detected at low levels and only in 50–75% of the samples. In contrast, a very high average hybridization signal was detected for *Arthroderma* in 95% of ER samples. In addition, high average hybridization signals were detected for *Penicillium*, *Rhizopus*, *Rhodotorula*, and *Cocciodes* in 80–90% of the TP samples, and *Arthroderma*, *Rhizopus*, and *Rhodotorula* in 80–97% of the HR samples (Fig. 3A and Supplementary Table S1).

Figure 3B represents prevalence of parasite genera in the four BC subtypes. The most diverse mycobiome was detected in ER+ and the least complex in TN, where all were detected at lower average hybridization signals (Fig. 3B and Supplementary Table S1). Among the parasites with higher detection, *Thelazia*, *Mansonella*, *Dirofilaria*, *Balantidium*, *Entamoeba*, and *Capillaria* were detected in greater than 90% of the ER samples; *Capillaria* and *Dirofilaria* in the TP samples; and *Thelazia* and *Dirofilaria* in over 93% of the HR samples (Fig. 3B and Supplementary Table S1).

Supplementary Fig. S1 shows a heatmap of the average hybridization signal for viruses and microorganisms detected in non-matched control tissues, matched control tissues, and the ER positive tumor tissues. Supplementary Figs. S2–S4 show similar heat maps for the TP, HR, and TN samples. In all the BC subtypes analyzed, we observed that the hybridization signals for the non-matched controls were significantly less intense than the tumor samples. Conversely, the hybridization signals for the matched control samples were more similar to the tumor samples. This is complicated by the finding that with hierarchical clustering there were sub-signatures for each subtype (Supplementary Fig. S5). However, this observation suggests that the tissue surrounding the tumor may take on biome characteristics that are similar to that of the tumor. Conversely, it suggests that a tumor-like microbiome maybe present on the breast tissues prior to tumor formation.

Supplementary Fig. S5 shows that each of the BC subtypes can be grouped into two or more sub-groups based on the higher, lower, or no detection of specific microbial signatures in their tumor microenvironment.

### Clinical correlations with the presence of specific microorganisms in TN breast cancer microbiome

**Fig. 4:**
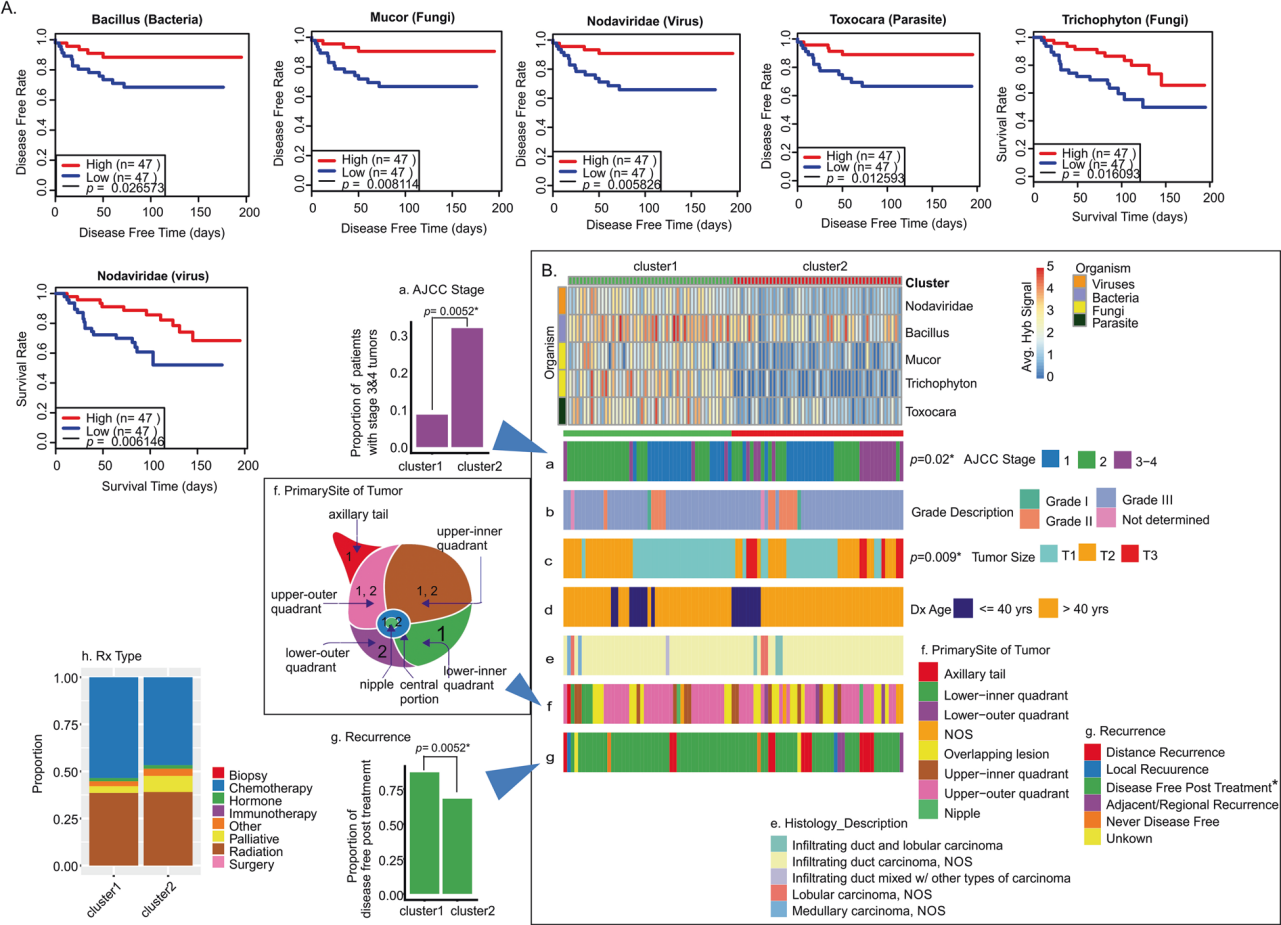
Oncobiome signatures in triple-negative breast cancer where higher hybridization signals correlated with better disease outcome. **A** The graphs show disease-free rate and/or survival relative to high or low hybridization signals for the specific microorganisms in the TN sample set. In the cases shown, higher hybridization signal correlates with increased disease-free time and/or survival. **B** TN BC samples were clustered based on high and low hybridization signals for those organisms where high hybridization signal correlated with higher disease-free time and survival (better outcomes). The high (cluster 1) and low (cluster 2) hybridization clusters were then correlated with clinical data shown as horizontal (**a**–**g**) and vertical (**h**) cluster barplot.

We next analyzed patient survival time or disease-free time post treatment with the presence of viruses and other microorganisms in the oncobiome. Analysis of TN patient samples showed higher average hybridization signals of *Bacillus*, *Mucor*, Nodaviridae, *Toxocara*, and *Trichophyton* that significantly correlated with longer disease-free time or survival time (Fig. 4A). Thus, we clustered the TN samples based on high and low hybridization signals for *Bacillus, Mucor*, Nodaviridae, *Toxocara*, and *Trichophyton* and correlated these clusters with clinical data (Fig. 4B and Supplementary Table S3). Note that not all samples originally tested had sufficient data on disease-free time and survival. We then compared the two resulting clusters (cluster 1: high hybridization signals; cluster 2: low hybridization signals) with clinical data [Fig. 4Ba (stage), 4Bb (grade), 4Bc (tumor size), 4Bd (age at diagnosis), 4Be (histology), 4Bf (Primary site of tumor), and 4Bg (recurrence)]. Among these we found significant differences in the two clusters related to stage, tumor size, recurrence, and position of the tumor in the breast. Specifically, patients in cluster 1 (high hybridization signal) had a much lower proportion of grade 2, 3, and 4 cancers (Fig. 4Ba), significantly smaller tumors (Fig. 4Bc), and a significantly longer disease-free period after treatment (Fig. 4Bg) compared to patients with a low hybridization signal (cluster 2). We also found that patients with TN tumors in the auxiliary tail and the lower inner quadrant of the breast were almost exclusively in cluster 1, while patients with tumors in the lower outer quadrant of the breast were almost exclusively in cluster 2. In Fig. 4Ch, we showed that the general treatment of patients in the two clusters was very similar; thus, the overall improved outcomes of patients in cluster 1 suggests that patients with higher hybridization signals for the five viruses and other microorganisms responded better to treatment.

Supplementary Fig. S6 shows additional microorganisms in the tumor oncobiome where higher average hybridization signals suggested a trend toward better disease prognosis for patients. In sum, these data suggest that the average hybridization signals of this subset of organisms in TN tumors can provide significant insights into the severity of the cancer and predictable outcomes.

### Clinical correlations with the presence of specific microorganisms in ER+ breast cancer microbiome

**Fig. 5:**
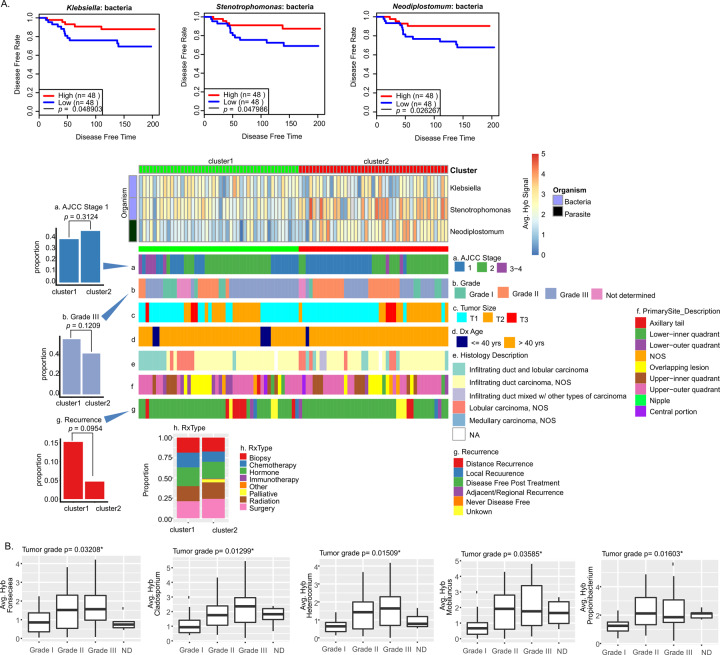
Oncobiome signatures in ER+ breast cancer where higher hybridization signals correlate with better disease outcome. **A** The graphs show disease-free rate relative to high or low hybridization signals for the specific microorganisms in the tumor sample set. In the cases shown, higher hybridization signal correlated with increased disease-free time or survival. The tumor samples were then clustered based on the hybridization levels for these microorganisms. The high (cluster 1) and the low (cluster 2) hybridization detection clusters were correlated with other clinical data shown as horizontal (**a**–**g**) and vertical (**h**) cluster barplot. **B** Box plot showing average hybridization signal of microorganism detection in different tumor grades. ND not diagnosed. *χ*^2^ *p* values showing significant (*p* ≤ 0.05) differences in the hybridization signal for detection in different grades provided.

We applied the same analysis to viruses and microorganisms in the ER cancer oncobiome. We detected higher average hybridization signals for two Proteobacteria (*Klebsiella* and *Stenotrophomonas*) and a parasite (*Neodiplostomum*) that significantly correlated with longer disease-free times post treatment (Fig. 5A). Thus, we clustered the ER samples based on low (cluster 1) and high (cluster 2) hybridization signals for these organisms, and correlated these clusters with all the clinical data available (Fig. 5Aa–g and Supplementary Table S4). We found significance differences between the two clusters for stage, grade, and recurrence. Specifically, the patients in cluster 2 (higher hybridization signals) tend to have: (1) a higher number of stage 1 cancer (Fig. 5Aa) and, correspondingly, a lower number of advanced stage 3 and 4 cancers; (2) a lower number of patients with Grade III cancer (Fig. 5Ab); and (3) a much lower proportion with distal recurrences post treatment (Fig. 5Ag). We also observed some differences in the general treatment provided for patients in the two clusters (Fig. 5Ah and Supplementary Table S3). For example, fewer patients in cluster 2 were given chemotherapy. This suggests that patients in the two clusters had a significantly different response to treatment.

Figure 5B shows additional analysis based on the correlation of tumor grade with the average hybridization signal for specific organisms. We observed higher detection of *Fonsecaea*, *Cladosporium*, *Heteroconium*, *Mobiluncus*, and *Propionibacterium* in grade 2 and 3 cancers (Fig. 5B).

Supplementary Fig. S7 shows ER tumor microorganisms that include bacterial genera *Bifidobacterium*, *Borrelia*, *Paracoccidioides*; fungal genera *Cunnighamella*; and parasitic genera of *Schistosoma*, *Plasmodium*, with higher average hybridization signals suggesting a trend toward improved disease prognosis for patients with ER BC subtype.

**Fig. 6:**
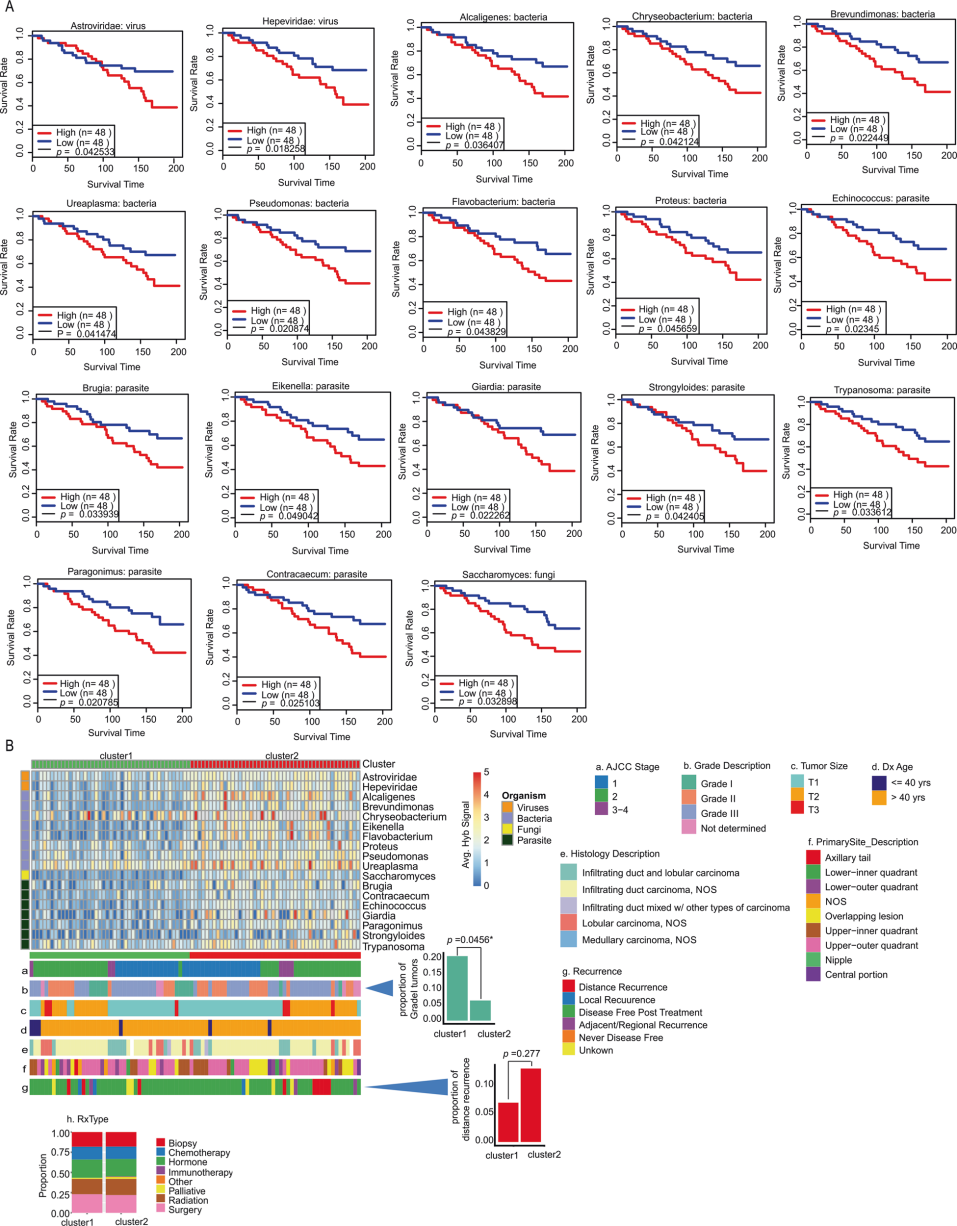
Oncobiome signatures in ER+ BCs where high hybridization signals correlated with worse disease outcome. **A** The graphs show survival time relative to high or low hybridization signals for the specific microorganisms shown. In the cases shown, higher hybridization signal correlated with decreased survival. **B**. ER+ BC samples were then clustered based on the hybridization levels for these microorganisms. The low (cluster 1) and the high (cluster 2) hybridization detection clusters correlated with other clinical data shown as horizontal (**a**–**g**) and vertical (**h**) cluster barplot.

Figure 6A shows that higher average hybridization signals in ER subtype for Astroviridae, Hepeviridae, Alcaligenes, Brevundimonas, Proteus, Eikenella, Pseudomonas, Chryseobacterium, Flavobacterium, Ureaplasma, Echinococcus, Giardia, Trypanosoma, Brugia, Strongyloides, Paragonimus, and Saccharomyces correlated with reduced survival rates. In Fig. 6B, the ER samples were clustered according to low (cluster 1) and high (cluster 2) hybridization signals for these organisms. We found that patients in cluster 2 tended to have: (1) a lower proportion of grade 1 tumors (Fig. 6Bb); and (2) and a higher proportion of distant recurrence of cancer post treatment (Fig. 6Bg). We did not find any significant differences in the treatment regime for the cluster 1 and 2 patients (Supplementary Table S5 and Fig. 6Bh) suggesting that cluster 2 patients may have responded better to specific treatments. Additional examples of microorganisms where higher detection may be associated with poor disease prognosis in ER+ cancer are shown in Supplementary Fig. S8.

As noted for TN cancers, the average hybridization signal of a small number of specific viruses and microorganisms in ER+ tumors can provide significant insight into the severity of the cancer and the predictable outcomes.

### Clinical correlations with the presence of specific microorganisms in HR tumors

**Fig. 7:**
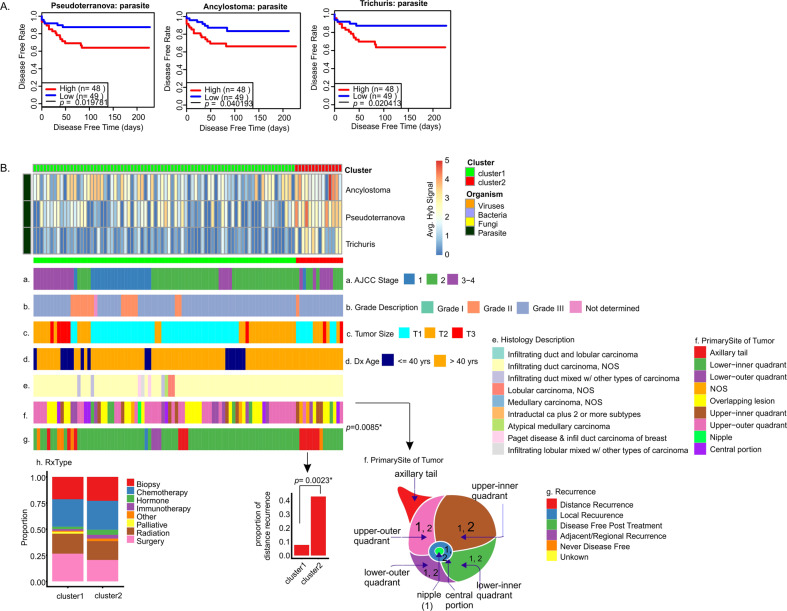
Oncobiome signatures in HER2+ BCs where high hybridization signals correlated with worse disease outcome. **A** The graphs show disease-free rate relative to high or low hybridization signals for the specific microorganisms in the tumor sample set. In the cases shown, higher hybridization signal correlates with decreased disease-free time. These microorganisms detected in patients with lower disease-free rate or survival were not significantly associated with higher cancer staging. **B** The tumor samples were then clustered based on the hybridization levels for these microorganisms. The low (cluster 1) and the high (cluster 2) hybridization detection clusters correlated with other clinical data shown as horizontal (**a**–**g**) and vertical (**h**) cluster barplot.

In HR cancers we did not find significant correlations between higher detection of specific microorganisms and better disease outcome, as indicated by less recurrence or greater survival (Supplementary Fig. S7). However, Fig. 7A shows that lower average hybridization signals for *Pseudoterranova*, *Ancylostoma*, *Trichuris*, and *Issatchenkia* statistically correlated with increased disease-free time after treatment. In Fig. 7B, we clustered the HR samples based on low (cluster 1) and high (cluster 2) hybridization signals for the four organisms. HR patients in cluster 2, who had relatively higher detection of these microorganisms (1) were mostly above 40 years of age (Fig. 7Bd); and (2) showed a higher proportion of distant recurrence of disease post treatment (Fig. 7Bg). There were no significant differences in the general treatment regime between cluster 1 and 2 (Fig. 7Bh and Supplementary Table S6) suggesting that patients in cluster 2 may not have responded to their treatment as well as patients in cluster 1. These data again suggested that the average hybridization signal of a few specific microorganisms in ER+ tumors can provide significant insights into the severity of the cancer and the predictable outcomes. However, a larger study may provide more statistical significance and thus broader and stronger correlations with available clinical data.

### Clinical correlations with the presence of specific microorganisms in TP tumors

**Fig. 8:**
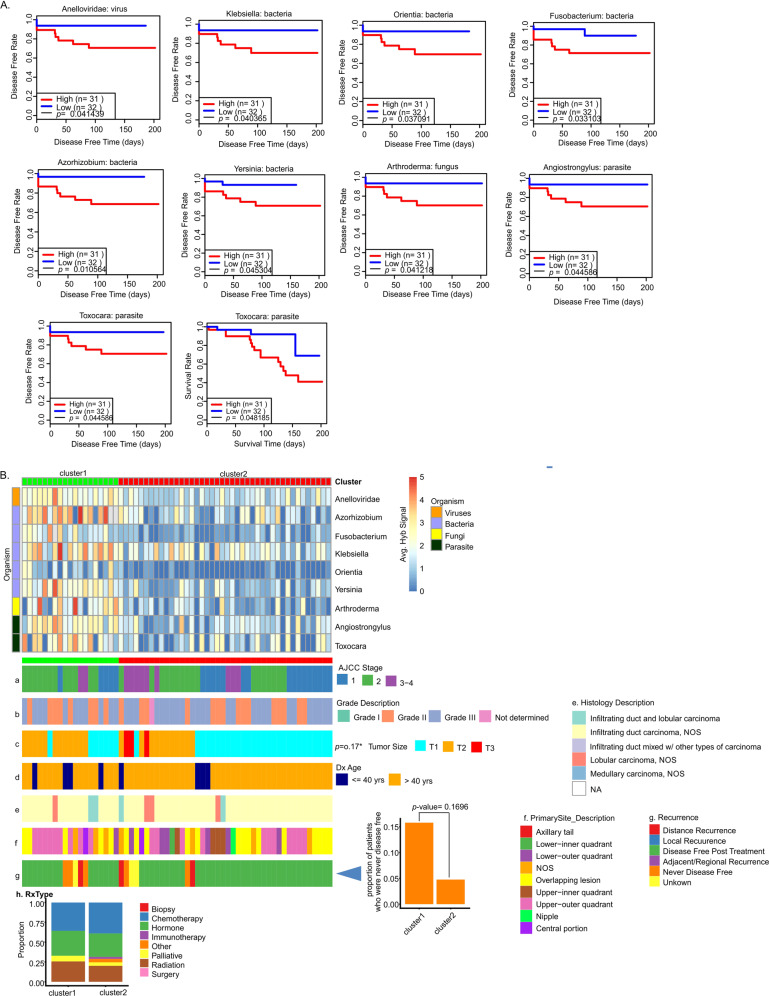
Oncobiome signatures in TP+ BCs where higher hybridization signals correlated with worse outcome. **A** The graphs show disease-free rate relative to high or low hybridization signals for specific microorganisms in the tumor sample set. In the cases shown, higher hybridization signal correlated with decreased disease-free time. **B** The tumor samples were then clustered based on the hybridization levels for these microorganisms. The high (cluster 1) and the low (cluster 2) hybridization detection clusters correlated with other clinical data shown as horizontal (**a**–**g**) and vertical (**h**) cluster barplot.

Analysis of the TP tumor data showed a number of organisms, where high average signals correlated significantly with less disease-free time post treatment or less survival time (Fig. 8A). These microorganisms include bacterial genera of *Orientia*, *Klebsiella*, *Fusobacterium, Azorhizobium*, *Yersinia, Arthroderma*, viral family Anelloviridae and parasitic genera *Angiostrongylus*, and *Toxocara*. Patients were clustered into high (cluster 1) and low (cluster 2) levels of detection and correlated with clinical data (Fig. 8B and Supplementary Table S7). The patients who had higher detection levels for these microorganisms (cluster 1) tended to have a higher proportion who were never disease free post treatment (Fig. 8Bg). However, this small number of patients in cluster 1 that resulted in this specific observation had limited statistical significance and so was strongly correlative. Supplementary Fig. S10 gives further examples of specific microorganism that tended to correlate with better or worse clinical outcome, but again for this group, the statistical significance was limited. A larger study of TP samples will increase sample size for the clusters and therefore increase the statistical significance and stronger correlations with clinical data.

## Discussions

We have previously studied the oncobiome of TN, ER, HR, and TP BC, the four subtypes of BC with a small number of study cohort [3]. In this current study, we re-examined our previous findings using an independent cohort of patients from a distinct site and screened over 400 BC samples, with associated clinical data. In agreement with the previous study, we showed that each BC subtype had a very diverse oncobiome with ER having the most diverse and TN having the least diverse oncobiome. There are many shared viruses and microorganisms across the oncobiomes, but also unique ones, the presence or absence of which can specifically distinguish TN, ER, HR, and TP cancers from each other. Since the four BC subtypes differ in Her2 expression and endocrine receptor signaling, it is possible that they have developed their own subtype-specific oncobiomes. Whether or not these oncobiomes contribute to the genesis or development of the cancers is still unknown. It is also possible that the tumor microenvironment provides a unique niche in which the components of the oncobiome can persist. No matter what the case may be, the unique oncobiomes provide biomarkers for diagnostic and prognostic purpose.

In this study we reported nucleic acid signatures of the viruses and other microorganisms that were found to be significantly higher in the BCs compared to healthy, non-matched controls (breast tissue from non-cancerous patients). The lack of detection of a specific virus or microorganism does not imply that the cancer is devoid of the virus or microorganism, but that the detection level is not significantly higher than the healthy non-matched controls. We found that matched control samples (pathologically normal tissues adjacent to the tumor tissue) often had microbial signatures that were significantly greater than the healthy, non-matched controls, and often similar to the levels seen in the tumors, most obvious in TN and ER cancers. This finding suggests two intriguing possibilities (1) that the oncobiome in the local microenvironment can extend to surrounding tissues; or (2) that the oncobiome found in the tumor formed prior to the genesis of the tumor. The latter possibility suggests a more active role at the site for tumor development. In this regard, we noted that the position of TN tumors in the breast correlated with levels of detection of specific oncobiome signatures.

The detection of nucleic acid signatures for different DNA viruses, such as herpesviruses, papillomaviruses, and polyomaviruses in different BC subtypes, has been well documented [6, 27–31]. What is more surprising is our consistent detection, in this and previous studies [3, 6], of signatures of poxviruses and parapoxviruses in the BC microbiome [32–35]. We have recently shown that the viral-VEGF encoded by Parapoxviruses, to promote proliferation of breast cancer and normal breast cells, while altering metabolic phenotype in normal breast cells, thus contributing to disease progression [36]. The detection of DNA signatures related to the Yaba Monkey Tumor Virus, a tumorigenic poxvirus, in all but TN cancer, suggests a potential role of this virus, or a human variant or fragments of this virus, in the oncogenic process [37]. Also noteworthy is the detection of mouse mammary tumor virus Env gene signatures significantly detected in all the BC types agreeing with our previous study and studies of others [6, 38, 39]. We cannot explain this finding but noted that not all probes for MMTV were detected and it may be that this represents a representation of a highly similar family member yet unidentified. In some BC subtypes that are not HIV positive, we noted significantly higher detection of signatures for HTLV, and other lentiviral signatures of SHIV, HIV-1, and bovine immunodeficiency virus 1 that may be uncharacterized human lentiviruses.

The abundance of the gram-negative Proteobacteria phylum detected tissues of the BC subtypes was not surprising, as it was reported earlier [3, 40–42], and may be associated with cancer development and/or with different responses to therapy. The detection of the signatures for the gram-negative anaerobic bacteria Fusobacterium in the BC subtypes was interesting as it is known to accelerate cancer development by enhancing cellular proliferation and protecting tumors from immune cell attack [43–45].

We detected signatures of skin fungi, yeasts, and parasitic signatures in all cancer types in agreement with our previous results [3]. In addition, the cancer samples contained signatures of previously described cancer-associated fungi such as *Fonsecaea*, *Trichosporon*, Microsporidians such as *Nosema* and *Pleistophora* [3, 6, 8, 46–49], and some parasites such as *Trypanosoma*, *Plasmodium*, *Strongyloides*, *Trichinella*, and *Taenia* [3, 6–8, 46, 50, 51].

Hierarchical clustering of the tumor microbiome signatures showed specific sub-signatures for each cancer, in agreement with previous studies [3, 6, 7, 18, 19, 52]. We examined whether the level of detection of viruses and other microorganisms strongly correlated with better or worse outcomes. As shown in Figs. 4–8, the levels of detection of a few viruses and microorganisms strongly correlated with survival time or disease-free time, and depended on the cancer subtype, tumor grade, tumor size, position of the tumor in the breast, and response to treatment. These data were statistically significant for TN and ER and strongly correlative for HR and TP. Thus, our data showed that the level of detection of some viruses and other microorganisms in the oncobiome of each BC subtype can provide significant prognostic and diagnostic value with insights into intervention strategies that can precisely target patients with a specific BC subtype.

Our study on more than 450 breast tumor samples, matched control, and non-matched control draws a comprehensive map showing the microbial population prevalent in each of the BC subtypes. We have successfully established a signature oncobiome for each BC subtype, and established a trend or correlation between the abundance of specific microbes with survival time or disease-free time for each subtype. Thus, our current study provides more clarity regarding the prognostic and diagnostic aspects of the oncobiome in BCs, which could be important for developing future treatment strategies with targeted precision therapies.

## Supplementary information

Supplementary Materials

Supplementary Figures

Supplementary Table S1

Supplementary Table S2

Supplementary Table S3

Supplementary Table S4

Supplementary Table S5

Supplementary Table S6

Supplementary Table S7

Supplementary Table S8

Supplementary Table S9

Supplementary Table S10
