# Supplementary material for: Prognostic correlations with the microbiome of breast cancer subtypes
Source: Cell Death Dis. 2021 Sep 4;12(9):831. doi: 10.1038/s41419-021-04092-x (PMC8418604; doi:10.1038/s41419-021-04092-x)
Supplement: Supplementary file 1 — Supplementary Materials [file 41419_2021_4092_MOESM1_ESM.docx]

**Supplementary Figure Legends**

Supplementary Figure S1. Heatmap of average hybridization signal of microbial detections in ER cancer, matched and non-matched controls.

Supplementary Figure S2. Heatmap of average hybridization signal of microbial detections in TP cancer, matched and non-matched controls.

Supplementary Figure S3. Heatmap of average hybridization signal of microbial detections in HR cancer, matched and non-matched controls.

Supplementary Figure S4. Heatmap of average hybridization signal of microbial detections in TN cancer, matched and non-matched controls.

Supplementary Figure S5. Hierarchical clustering of the 4 breast cancer types based on the microbial signature detections.

Supplementary Figure S6. Higher detection of certain micro-organisms in TN breast cancer patients tend to have higher disease free time and/or survival time.

Supplementary Figure S7. Higher detection of certain micro-organisms in ER positive breast cancer patients tend to have higher disease free time and/or survival time.

Supplementary Figure S8. Higher detection of certain micro-organisms in ER positive breast cancer patients tend to have lower disease free time and/or survival time.

Supplementary Figure S9. Higher detection of certain micro-organisms in HR positive breast cancer patients’ trend towards having an either a better or worse disease outcome.

**Supplementary table legends**

Supplementary table S1. Average hybridization signal and prevalence of micro-organism signatures detected in different breast cancer types. Each individual tabs of the excel sheet represents the data for viral, bacterial, fungal and parasitic signatures respectively.

Supplementary table S2. The differences in microbial detections between different types of breast cancers that are grouped by topological analysis.

Table S3-S7. Statistics of different clinical information (diagnosis, ages, grades, histology) in each cluster identified by the organisms correlated with disease outcomes for each breast cancer types.

Supplementary table S8. The Kaplan-Meier and cox regression analysis of survival and disease free rates of organisms in different types of breast cancer.

Supplementary table S9. Cox regression between survival or disease free time and clinical covariates in four types of breast cancers.

Supplementary Table S10. Viral signatures detected in 4 breast cancer types
