# Supplementary material for: Prognostic correlations with the microbiome of breast cancer subtypes
Source: Cell Death Dis. 2021 Sep 4;12(9):831. doi: 10.1038/s41419-021-04092-x (PMC8418604; doi:10.1038/s41419-021-04092-x)

Supplementary Figure S1

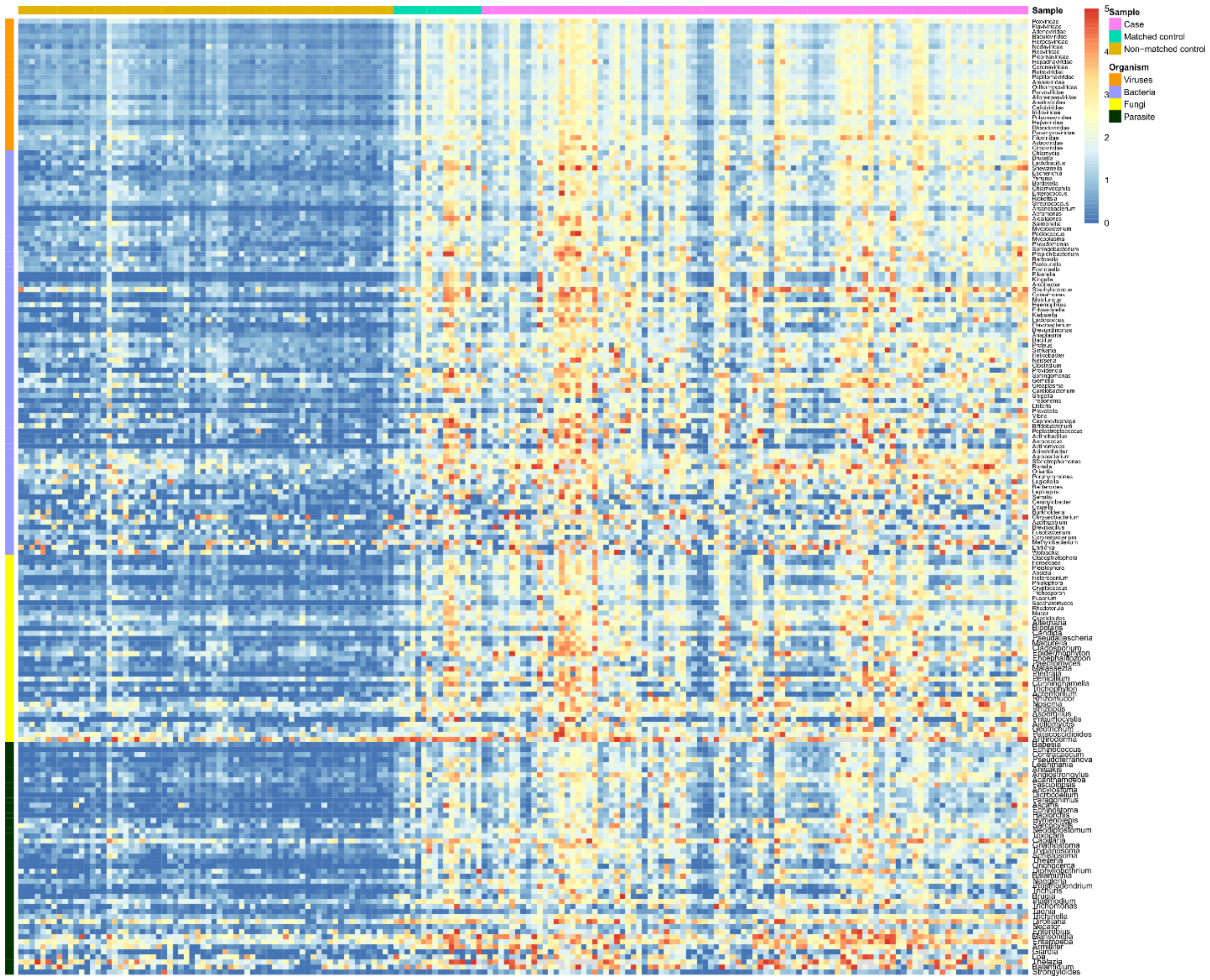

Supplementary Figure S2

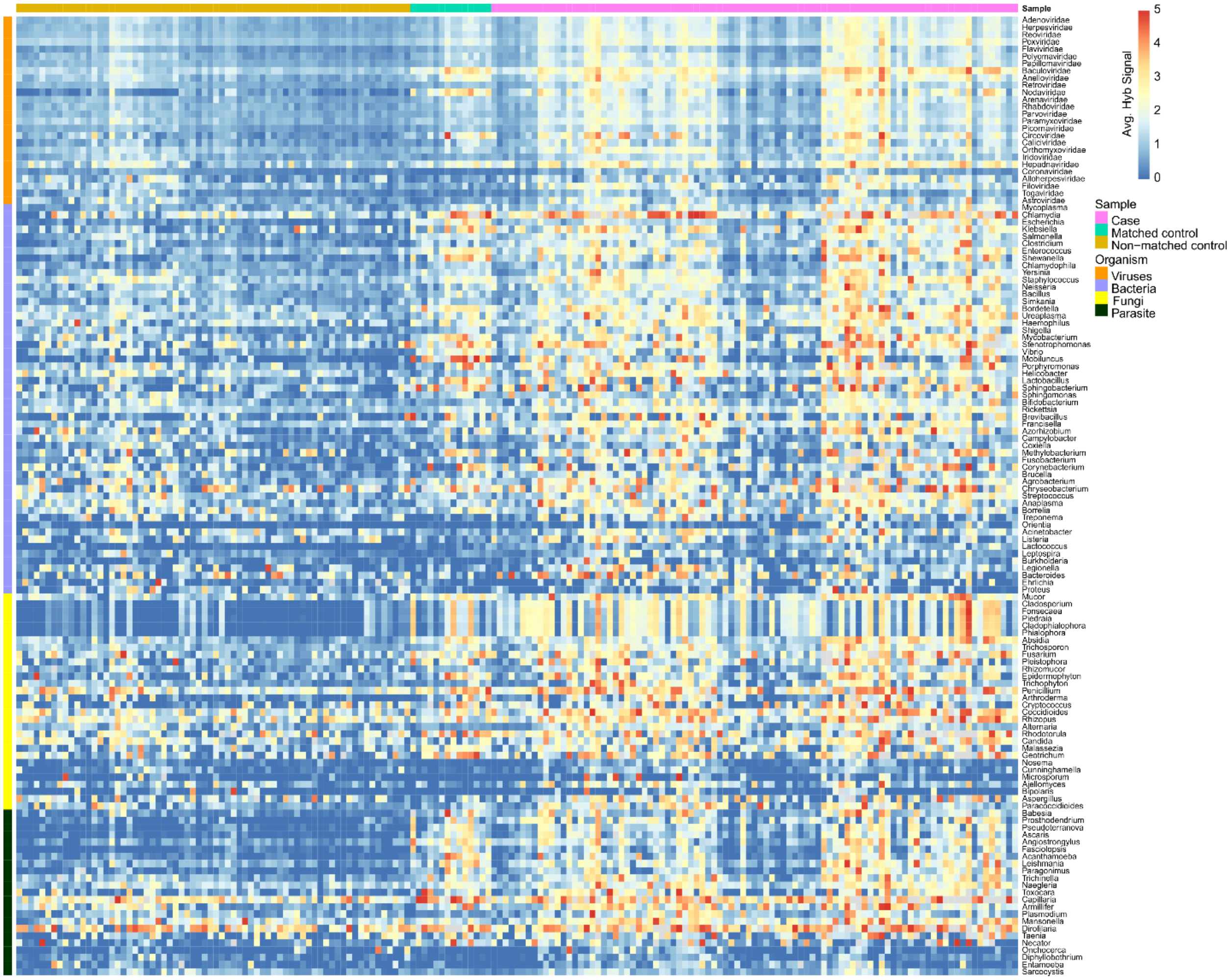

Supplementary Figure S3

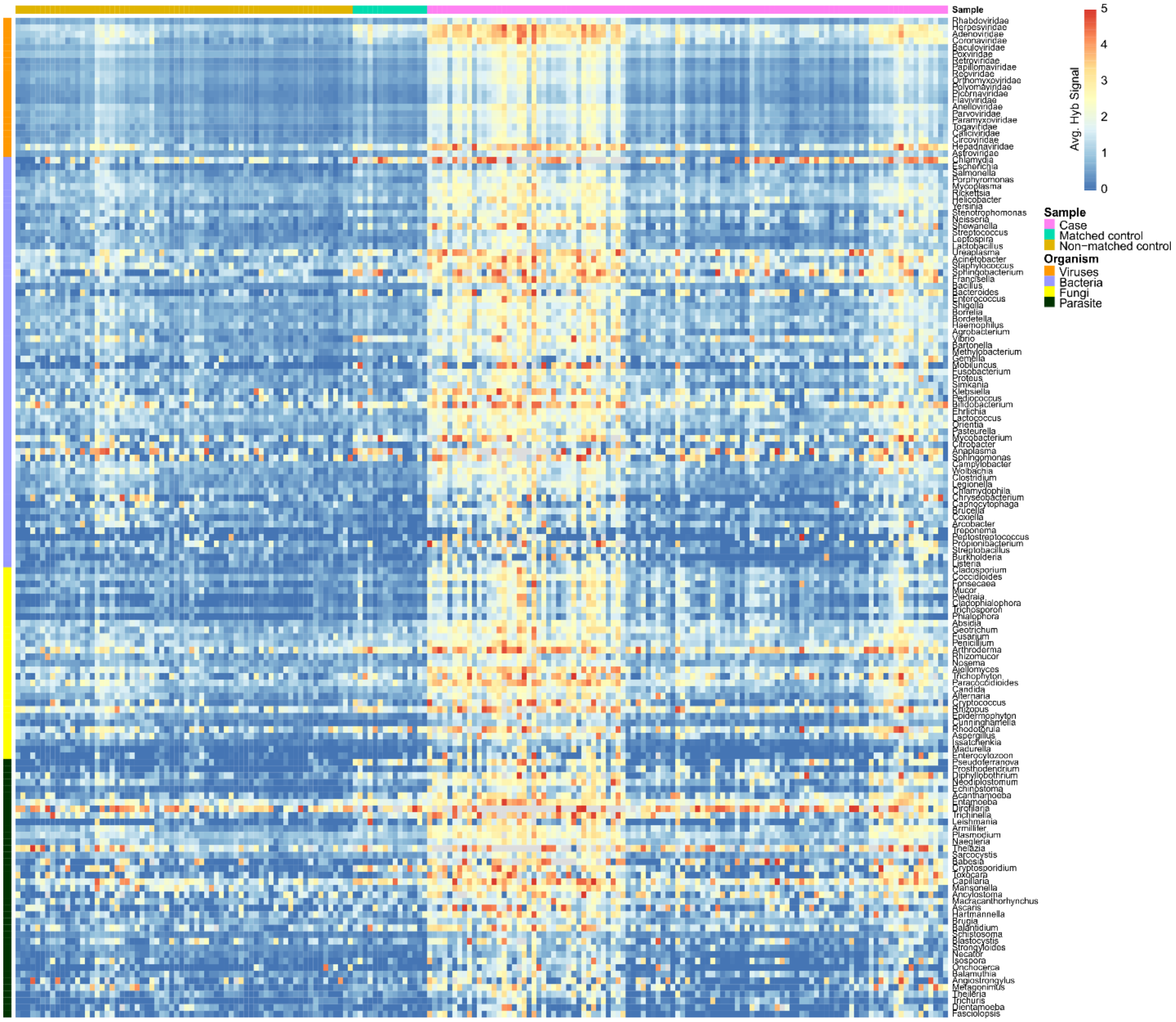

Supplementary Figure S4

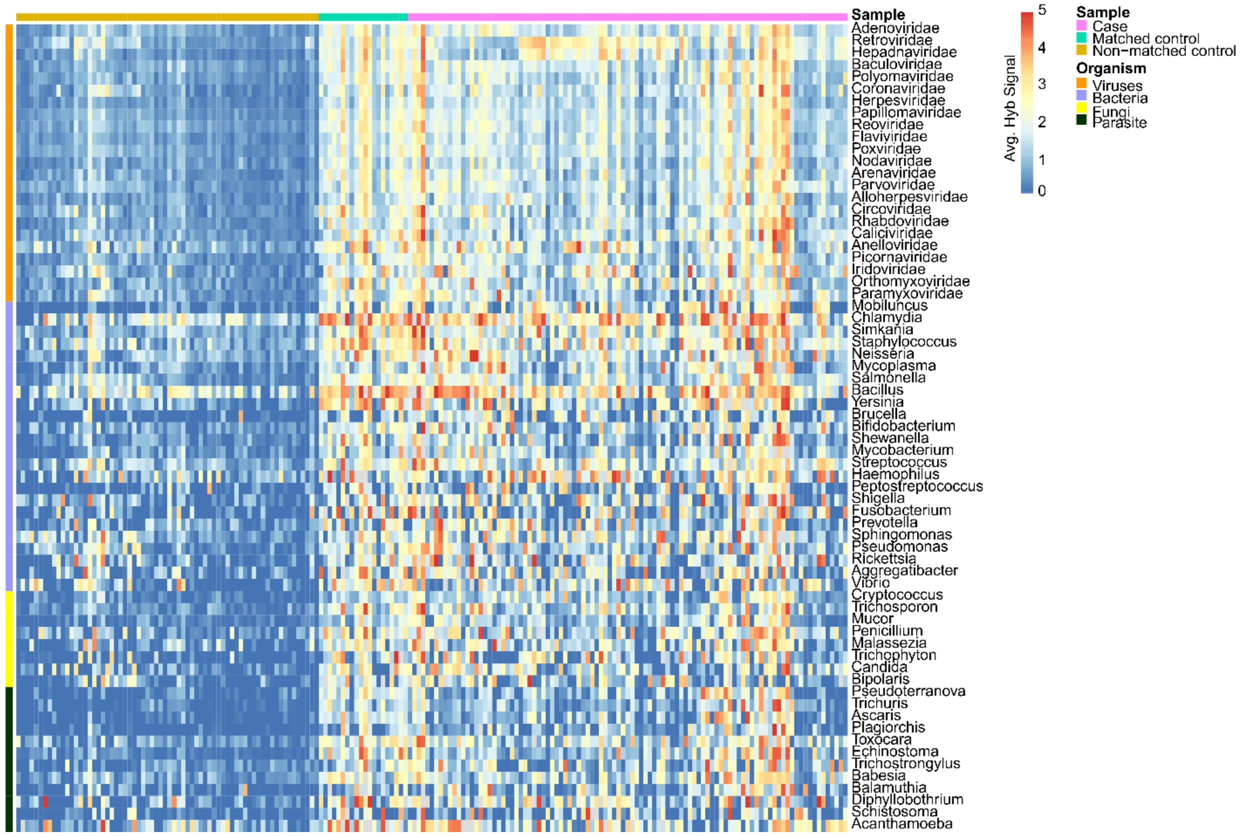

Supplementary Figure S5

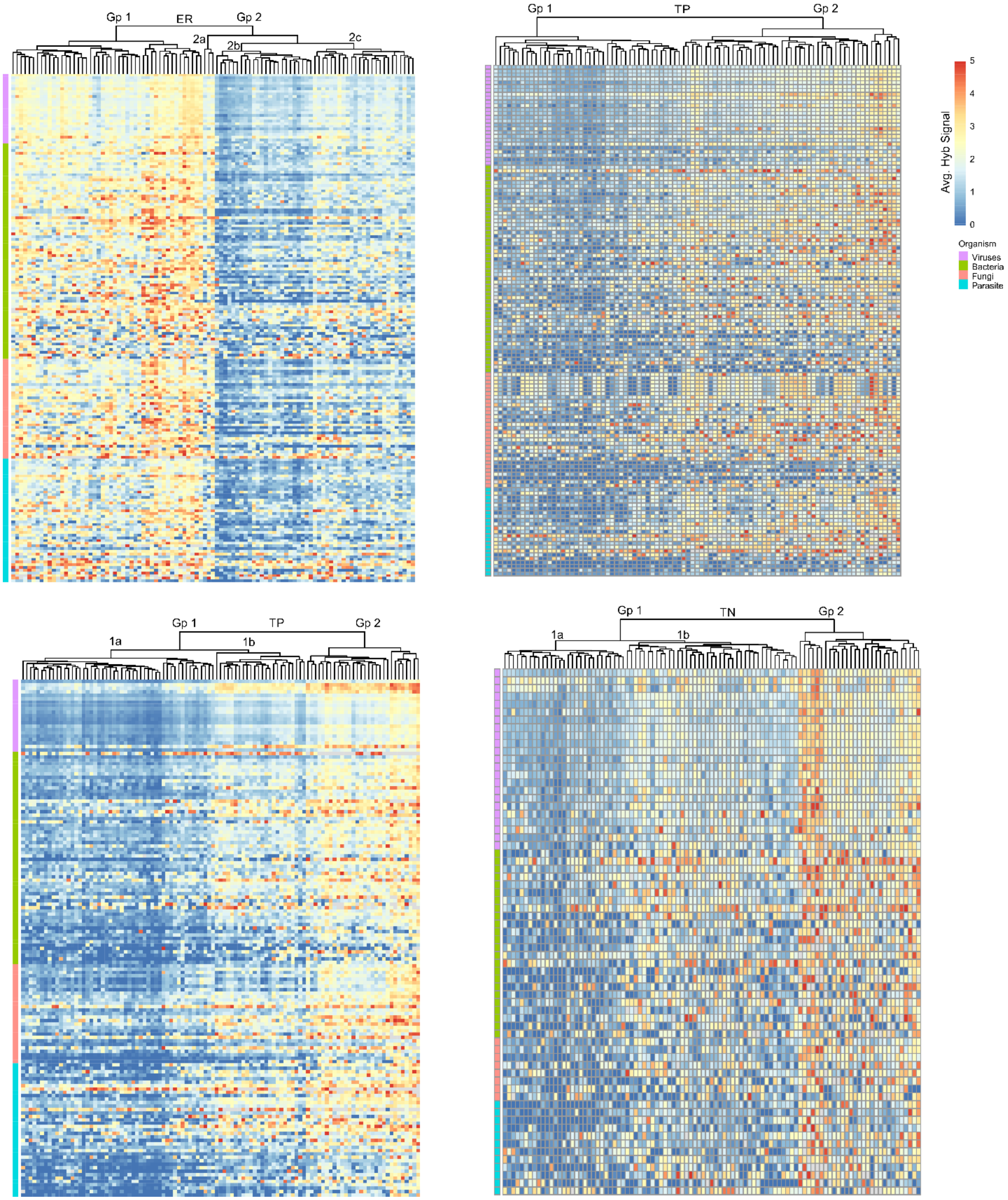

Supplementary Figure S6

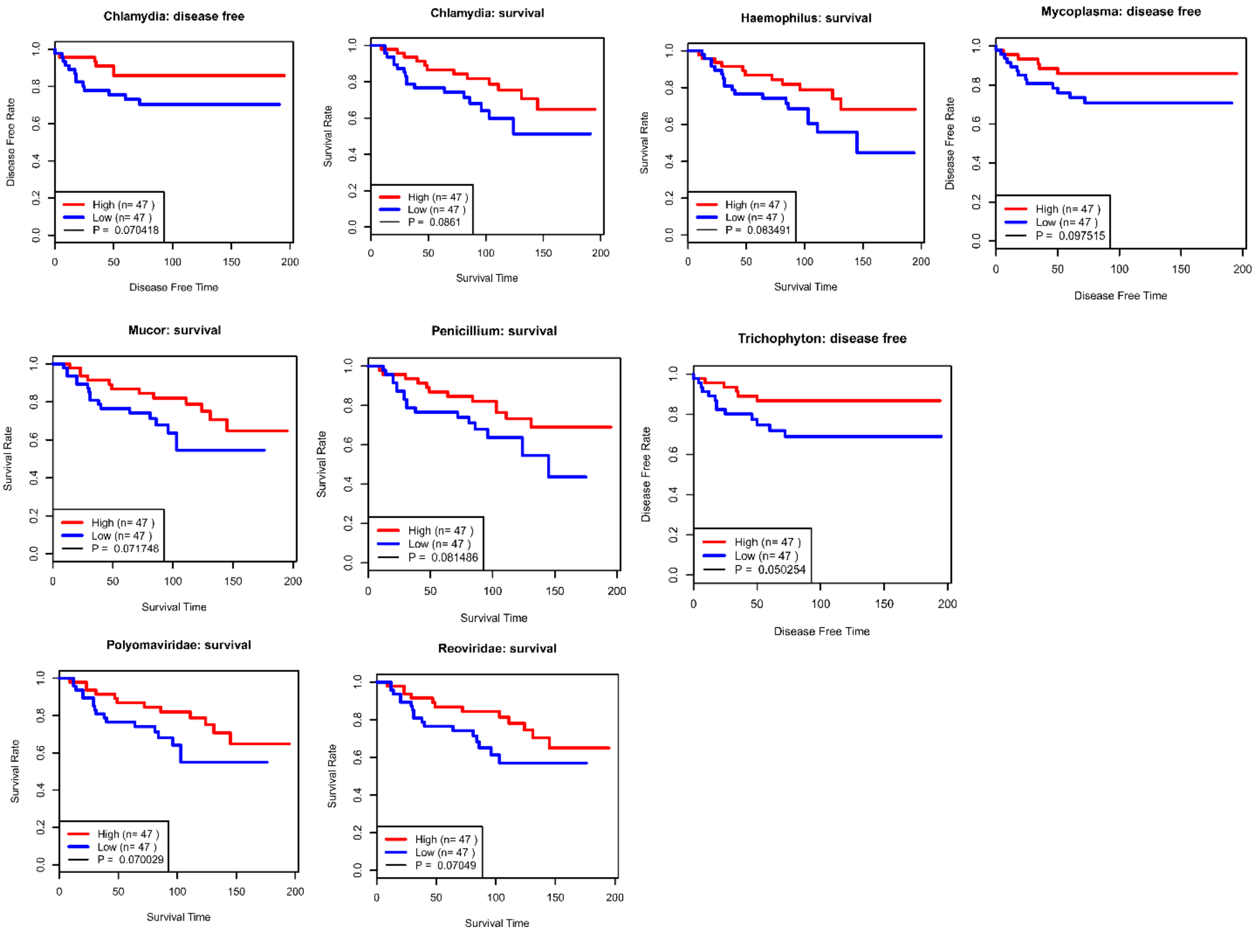

Supplementary Figure S7

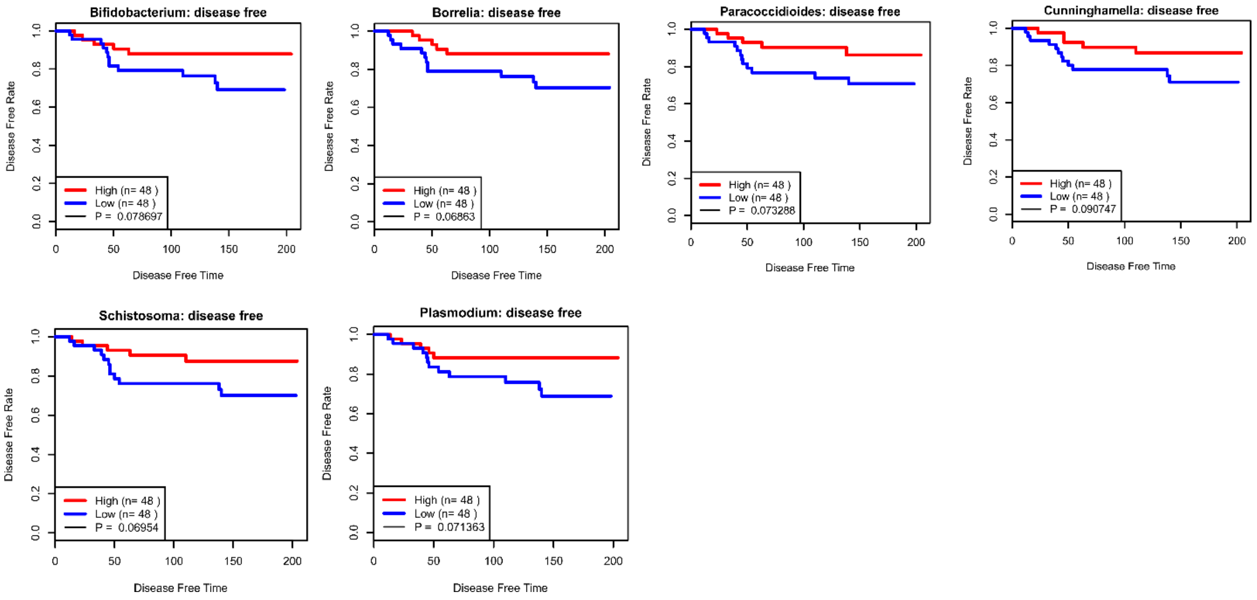

Supplementary Figure S8

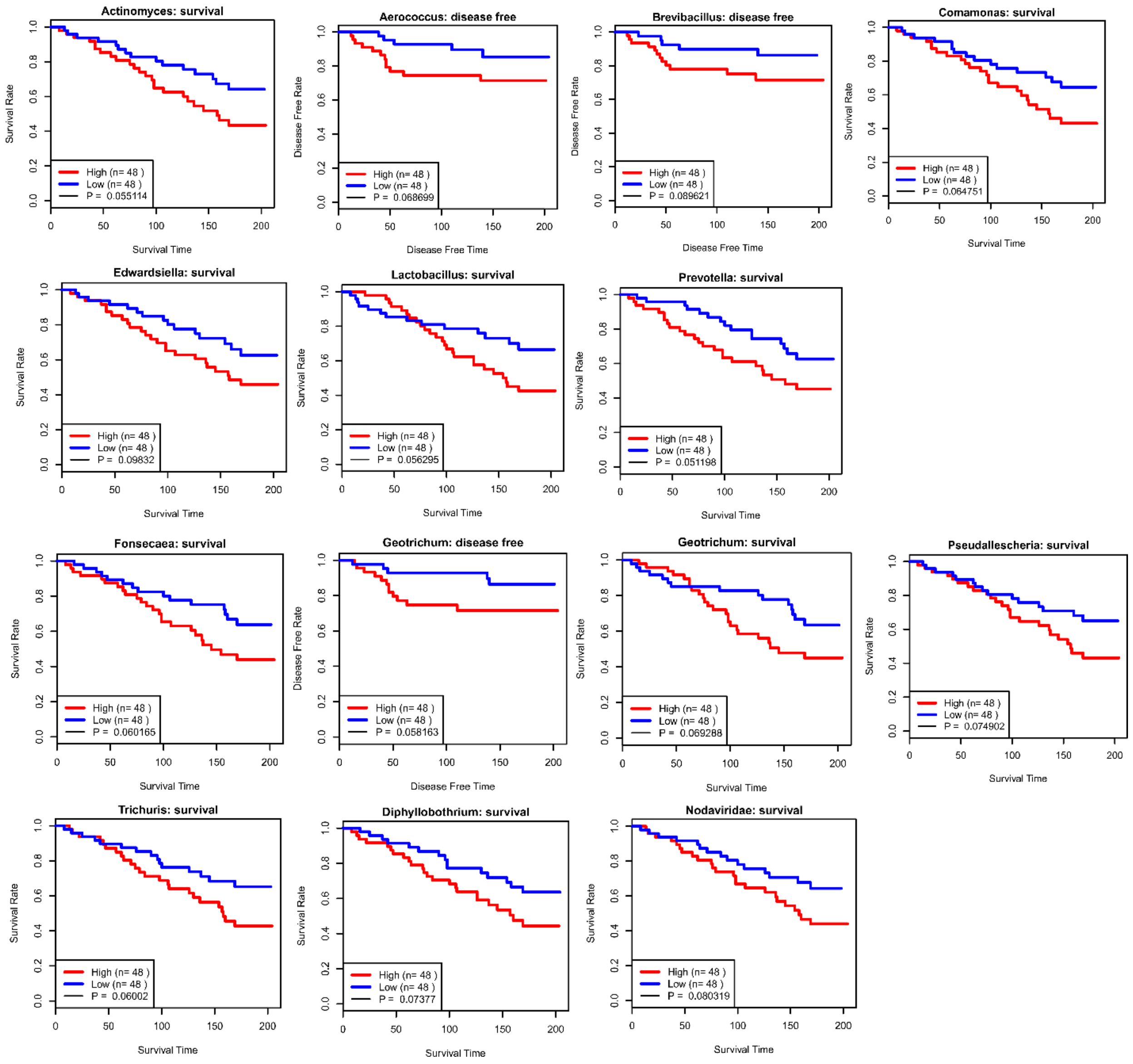

Supplementary Figure S9

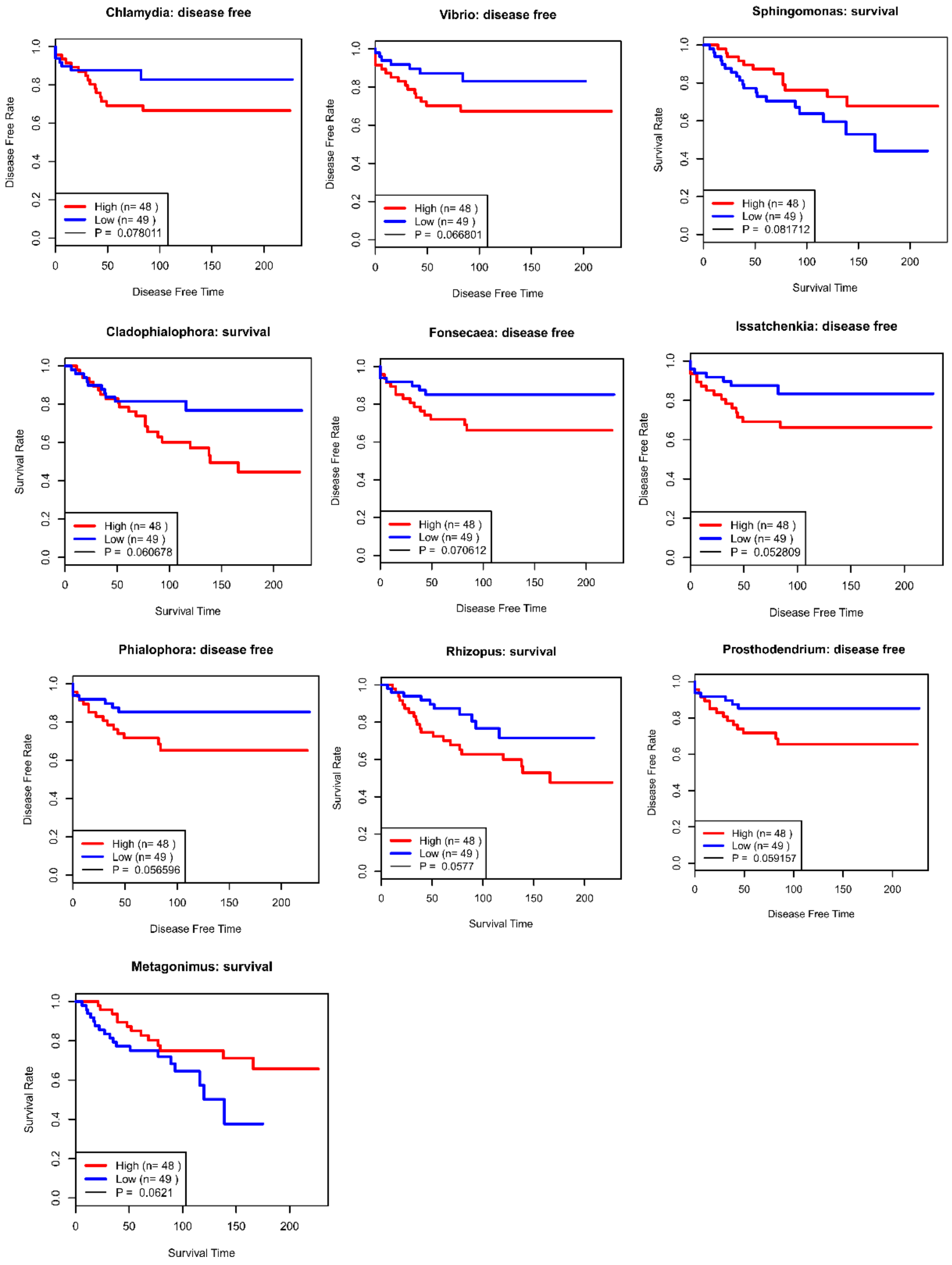

Supplementary Figure S10

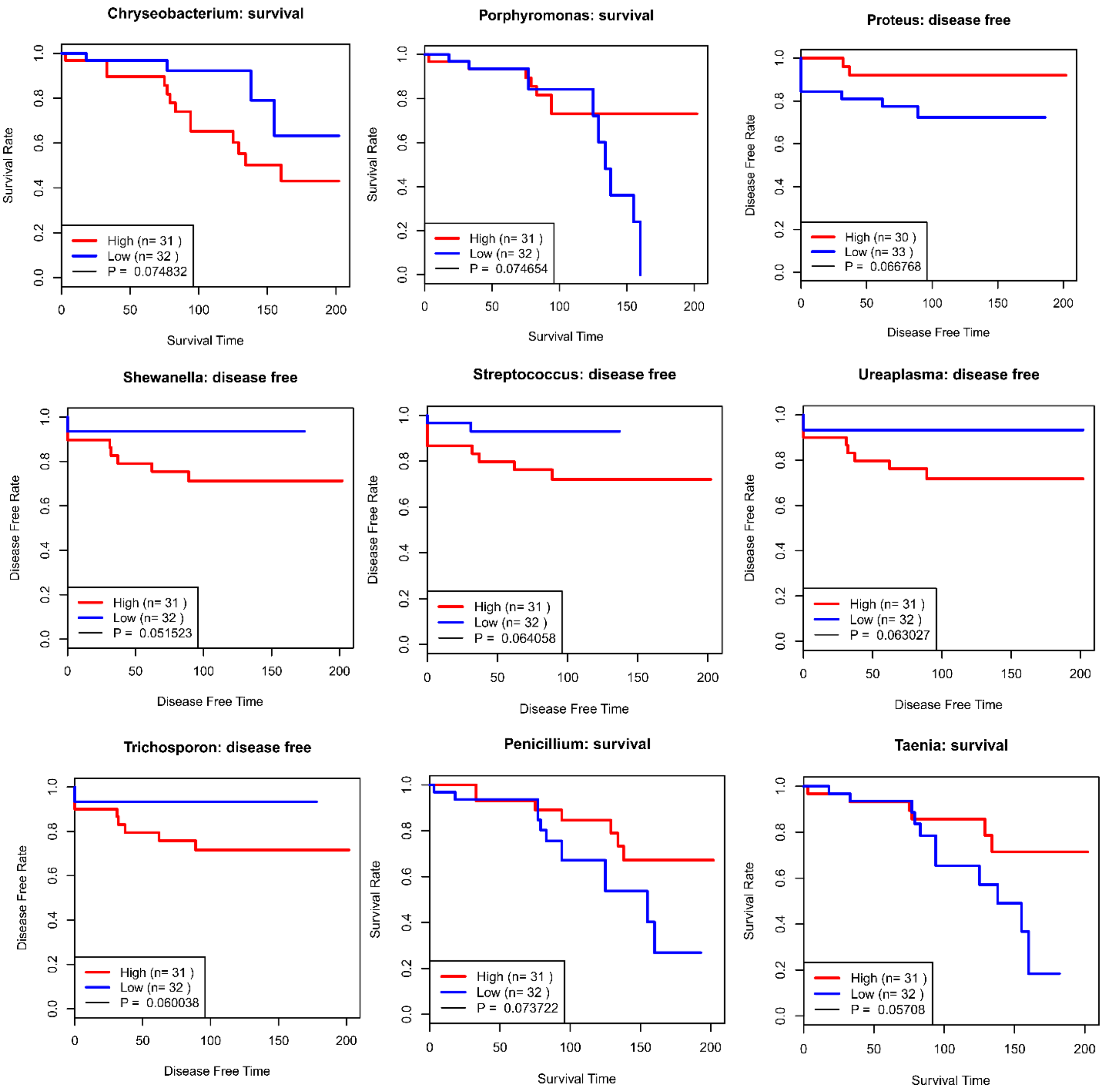

Supplement: Supplementary file 2 — Supplementary Figures [file 41419_2021_4092_MOESM2_ESM.pdf]
