# Supplementary material for: Prognostic correlations with the microbiome of breast cancer subtypes
Source: Cell Death Dis. 2021 Sep 4;12(9):831. doi: 10.1038/s41419-021-04092-x (PMC8418604; doi:10.1038/s41419-021-04092-x)
Supplement: Supplementary file 12 — Supplementary Table S10 [file 41419_2021_4092_MOESM12_ESM.docx]

Table 1. Viral signatures detected in 4 breast cancer types

| Cancer.type | Viral Signatures | Bacterial Signatures | Fungal Signatures | Parasitic Signature |
| --- | --- | --- | --- | --- |
| ER | Hepeviridae | *Arcanobacterium, Aeromonas, Alcaligenes, Eikenella, Kingella, Comamonas, Edwardsiella, Flavobacterium, Brevundimonas, Providencia, Cardiobacterium, Actinobacillus, Aerococcus, Actinomyces, Serratia* | *Heteroconium, Saccharomyces, Pseudallescheria, Encephalitozoon, Paecilomyces, Acremonium, Pneumocystis* | *Echinococcus, Contracaecum, Anisakis, Dicrocoelium, Haplorchis, Hymenolepis, Gnathostoma, Trypanosoma, Trichomonas, Enterobius, Giardia, Loa* |
| TP |  |  | *Microsporum* |  |
| HR |  | *Citrobacter, Streptobacillus* | *Issatchenkia, Enterocytozoon* | *Cryptosporidium, Macracanthorhynchus, Hartmannella, Blastocystis, Isospora, Metagonimus, Dientamoeba* |
| TN |  | *Aggregatibacter* |  | *Plagiorchis, Trichostrongylus* |
| ER:TP | Filoviridae | *Azorhizobium, Brevibacillus, Corynebacterium* | *Pleistophora* | *Paragonimus, Taenia* |
| HR:TP | Togaviridae |  |  |  |
| ER:HR |  | *Pediococcus, Propionibacterium, Bartonella, Pasteurella, Arcobacter, Gemella, Capnocytophaga, Wolbachia* | *Madurella* | *Ancylostoma, Neodiplostomum, Theileria, Brugia, Thelazia, Balantidium, Strongyloides* |
| ER:TN |  | *Pseudomonas, Prevotella* |  |  |
| ER:HR:TP | Astroviridae | *Lactobacillus, Escherichia, Bordetella, Chlamydophila, Enterococcus, Sphingobacterium, Francisella, Klebsiella, Lactococcus, Anaplasma, Proteus, Helicobacter, Clostridium, Ureaplasma, Treponema, Listeria, Acinetobacter, Agrobacterium, Stenotrophomonas, Borrelia, Orientia, Porphyromonas, Legionella, Bacteroides, Leptospira, Campylobacter, Coxiella, Burkholderia, Chryseobacterium, Methylobacterium, Ehrlichia* | *Cladophialophora, Fonsecaea, Absidia, Phialophora, Fusarium, Rhodotorula, Coccidioides, Alternaria, Cladosporium, Epidermophyton, Piedraia, Cunninghamella, Rhizomucor, Nosema, Rhizopus, Aspergillus, Ajellomyces, Geotrichum, Paracoccidioides, Arthroderma* | *Leishmania, Angiostrongylus, Fasciolopsis, Sarcocystis, Capillaria, Onchocerca, Naegleria, Prosthodendrium, Plasmodium, Trichinella, Dirofilaria, Necator, Mansonella, Entamoeba, Armillifer* |
| ER:HR:TN |  | *Peptostreptococcus* |  | *Echinostoma, Schistosoma, Balamuthia, Trichuris* |
| ER:TN:TP | Nodaviridae, Arenaviridae, Alloherpesviridae, Iridoviridae |  | *Bipolaris, Malassezia* |  |
| ER:HR:TN:TP | Poxviridae, Flaviviridae, Adenoviridae, Baculoviridae, Herpesviridae, Reoviridae, Picornaviridae, Hepadnaviridae, Coronaviridae, Retroviridae, Papillomaviridae, Orthomyxoviridae, Parvoviridae, Anelloviridae, Caliciviridae, Polyomaviridae, Rhabdoviridae, Paramyxoviridae, Circoviridae | *Chlamydia, Brucella, Shewanella, Yersinia, Rickettsia, Streptococcus, Salmonella, Mycobacterium, Mycoplasma, Staphylococcus, Mobiluncus, Haemophilus, Bacillus, Simkania, Neisseria, Sphingomonas, Shigella, Vibrio, Bifidobacterium, Fusobacterium* | *Cryptococcus, Trichosporon, Mucor, Candida, Penicillium, Trichophyton* | *Babesia, Pseudoterranova, Acanthamoeba, Ascaris, Toxocara, Diphyllobothrium* |
